# Cost Effective Silver Nanowire-Decorated Graphene Paper for Drop-On SERS Biodetection

**DOI:** 10.3390/nano11061495

**Published:** 2021-06-04

**Authors:** Chiara Amicucci, Cristiano D’Andrea, Marella de Angelis, Martina Banchelli, Roberto Pini, Paolo Matteini

**Affiliations:** 1 “Nello Carrara” Institute of Applied Physics (IFAC), Italian National Research Council (CNR), Via Madonna del Piano 10, 50019 Sesto Fiorentino, Italy; chiara.amicucci@unifi.it (C.A.); c.dandrea@ifac.cnr.it (C.D.); m.deangelis@ifac.cnr.it (M.d.A.); m.banchelli@ifac.cnr.it (M.B.); r.pini@ifac.cnr.it (R.P.); 2 Department of Industrial Engineering, University of Florence, Via Santa Marta 3, 50134 Florence, Italy

**Keywords:** surface-enhanced Raman scattering (SERS), plasmonic nanoparticles, biomolecules detection, disposable SERS substrates, near field

## Abstract

The use of SERS for real-world bioanalytical applications represents a concrete opportunity, which, however, is being largely delayed by the inadequacy of existing substrates used to collect SERS spectra. In particular, the main bottleneck is their poor usability, as in the case of unsupported noble metal colloidal nanoparticles or because of the need for complex or highly specialized fabrication procedures, especially in view of a large-scale commercial diffusion. In this work, we introduce a graphene paper-supported plasmonic substrate for biodetection as obtained by a simple and rapid aerosol deposition patterning of silver nanowires. This substrate is compatible with the analysis of small (2 μL) analyte drops, providing stable SERS signals at sub-millimolar concentration and a detection limit down to the nanogram level in the case of hemoglobin. The presence of a graphene underlayer assures an even surface distribution of SERS hotspots with improved stability of the SERS signal, the collection of well-resolved and intense SERS spectra, and an ultra-flat and photostable SERS background in comparison with other popular disposable supports.

## 1. Introduction

Two-dimensional arrays of plasmonic nanoparticles are gaining consensus within the scientific community in surface-enhanced Raman scattering (SERS) detection [1,2] of molecules of interest in life science, thanks to their easy handling and reduced signal variability as compared to traditionally used unsupported colloidal particles. In particular, functional SERS substrates obtained by low cost, rapid, and simple fabrication methods, such as by micropipetting [3,4,5,6], ink-jet [7,8], screen-printing [9,10], and filtration [11,12] of plasmonic nanoparticles are becoming appealing tools in view of promoting SERS to the level of an accepted and sustainable option for every-daily life applications or routine basic research [13]. Nonetheless, the technological solutions proposed so far have been scarcely addressed in the practical detection of molecules of biomedical significance, especially in view of their use close to or near the point-of-need settings and of sustainable production and commercial exploitation. We recently introduced a disposable SERS substrate in the form of a spotted membrane of silver nanowires (AgNWs), as obtained by a combined bottom-up/top-down scheme based on the flow-through method, plus laser patterning for rapid label-free analysis of small volumes of biological species [12]. This system provided a successful detection of proteins with different molecular weights, hydrodynamic radius, and secondary structures [14] and was tested in the chemostructural discrimination between toxic and nontoxic amyloid beta forms of Alzheimer’s disease [15].

In this work, we introduce a highly responsive SERS substrate specifically designed for biodetection of small sample drops and aimed at overcoming some limitations of the previous system as a result of the use of a simplified fabrication procedure and the introduction of a graphene underlayer to improve the SERS response. On the one hand, the fabrication setup was implemented with a common nebulizer for aerosol therapy, assuring a facile deposition of AgNWs without the need for expensive, time-consuming, or highly specialized procedures. Several studies proposed the direct deposition of sprayed colloids on sample surfaces to probe the surface composition of atmospheric particles [16], inks, and colorants in historic documents [17] or pesticides in fruits [18,19]. On the other hand, the preparation of 2D plasmonic substrates by sprayed or nebulized nanoparticles and their use within SERS assays for the determination of trace substances remains an underexplored field, so far [20,21,22,23,24]. Another feature of the proposed substrate deals with the use of graphene paper to host the nanoparticle deposits, offering a flat background and imparting superior reproducibility. The use of graphene as a support for plasmonic nanostructures to improve SERS signal detection has become a popular choice within the SERS community in recent years. Several excellent groundworks have been published in this field, demonstrating remarkable benefits offered by the introduction of a graphene sublayer once the resulting hybrid systems are tested against small model and organic analytes [25,26,27,28,29]. However, the demonstration of a real efficacy of these systems in the analysis of molecular species of biological/biomedical interests still represents a challenging gap to overcome before accepting them as effective tools in everyday life applications.

## 2. Materials and Methods

### 2.1. Chemicals

Polyvinylpirrolidone (PVP, M_w_ 40000), isopropanol (99.5%), myoglobin from horse skeletal muscle (Mb), and hemoglobin (Hb) were purchased from Sigma-Aldrich (St. Louis, MO, USA). Ethylene glycol (EG, 99%) was purchased from Carlo Erba (Milan, Italy). Silver nitrate (AgNO_3_) and silver chloride (AgCl) were obtained from Cabro S.p.A. (Arezzo, Italy).

### 2.2. AgNWs Synthesis

AgNWs were synthesized by the polyol method. Briefly, 80 mL of EG was heated and thermally stabilized at 170 °C in a flask. Once the temperature had been stabilized, 112.5 mg of AgCl was added to the flask. Meanwhile, 495 mg of AgNO_3_ and 3 g of PVP were dissolved in 10 mL of EG each. The PVP and AgNO_3_ solutions were poured into two 10 mL syringes, which were placed in a syringe pump. Three minutes after the addition of AgCl, the slow injection of the two reagents was started with an injection rate of 0.5 mL/min. The reaction proceeded until the injection was finished. Afterward, the flask was cooled in an ice bath. The suspension was poured in 600 mL of acetone to leave AgNWs to spontaneously settle down overnight. Supernatant was removed and AgNWs were re-dispersed in isopropyl alcohol. AgNWs were characterized by SEM (Zeiss, EVO MA 10, Jena, Germany) and UV-Vis spectroscopy (PerkinElmer Lambda 35 UV/Vis, Norwalk, CT, USA). The concentration of the as-obtained AgNWs suspension was finally determined as 1 mg/L by gravimetric determination.

### 2.3. Fabrication of SERS Substrates

SERS substrates were fabricated by aerosol deposition of AgNWs on 50-μm thick graphene-based paper (G2Nan Sheet 50, Nanesa S.r.l.) as achieved by mechanical compression of small stacks of graphene, which in turn was obtained by exfoliation of expanded graphite. Different AgNWs dispersions, as obtained by sequential dilutions in isopropyl alcohol of the original solution, were aerosolized on varying the exposition time within the 1–15 min range by using a common compressor nebulizer emitting micron-sized AgNWs drops for aerosol therapy (Master-Aid Dynamic Aerosol, Pietrasanta Pharma S.p.A., Capannori, Italy). A fixed spacing between the nebulizer output and the graphene paper was maintained by using a third-hand support clip. Before deposition, the graphene was wetted in ethanol and then adhered to a PET mask (Melinex^®^ 454 polyester film, thickness 125 µm, DuPont, Wilmington, DE, USA ) patterned with 1.5-mm in size round holes, obtained by mechanical punching. 

The overall distribution of deposited AgNWs on graphene paper was investigated by optical microscopy (OM) (Olympus, BX41, Tokyo, Japan). The morphology of AgNWs on graphene paper at the nanoscale was analyzed by tapping mode AFM by using a JPK NanoWizard III Sense (Berlin, Germany) scanning probe microscope at a 250–300 kHz drive frequency and a 0.5 Hz scan rate and equipped with single-beam uncoated silicon cantilevers (μMash HQ:NSC15 Cr-Au BS).

### 2.4. SERS Measurements

The as-fabricated substrates were analyzed using a micro-Raman spectrometer (XPlora, Horiba, Kyoto, Japan) working at 532 nm with 1200 grooves/mm grating, an integration time of 5 s, and laser power at the sample of 150 μW, unless otherwise specified. A 10× objective with 0.25 NA (7 μm waist) was used.

### 2.5. FEM Simulation

The electric field distribution in the near proximity of the AgNWs had been evaluated using a commercial FEM package, the wave optical module of COMSOL multiphysics (Stockholm, Sweden, v 5.1), and the MNPBEM MATLAB (Natick, MA, USA) toolbox for the simulation of metallic nanoparticles, using a boundary element method approach [30].

We chose to depict the AgNW as a cylinder with hemispherical ends, a radius of 25 nm, a total length 5 μm, and a refractive index for silver taken from Rakić et al. [31]. An evaluation of the different arrangements established by the nanowires on the substrate was done based on AFM analysis, revealing that the nanowires laid down in contact with the substrate surface and most of them experienced a cross intersection with other wires. That is, we estimated, on average, a number of intersections that were at least 80% of the total number of wires deposited. This is why for our simulation we considered two configurations. In the first one, a AgNW was lying on a substrate with the index of refraction of graphene from Zhu et al. [32]. In the second one, a AgNW was in air, verifying the proximity of a second crossing wire. In both cases, AgNWs were illuminated by a 532 nm plane wave from above and the calculation of the electric field |E|/|E_0_| values were averaged over 5 different polarization angles of the incident light. A further configuration including a single AgNW laying on graphene and surmounted by another crossed wire was presented in the Supplementary Materials to exclude a significant mutual influence between adjacent hotspots. In general, incident light polarized the ends and gave rise to a standing surface charge wave propagating along the wire [33]. In the electric field spectrum, nanowires typically exhibited several higher-order modes within the visible spectral region due to their significant dimensions with respect to the illumination wavelength [34].

## 3. Results and Discussion

**Figure 1:**
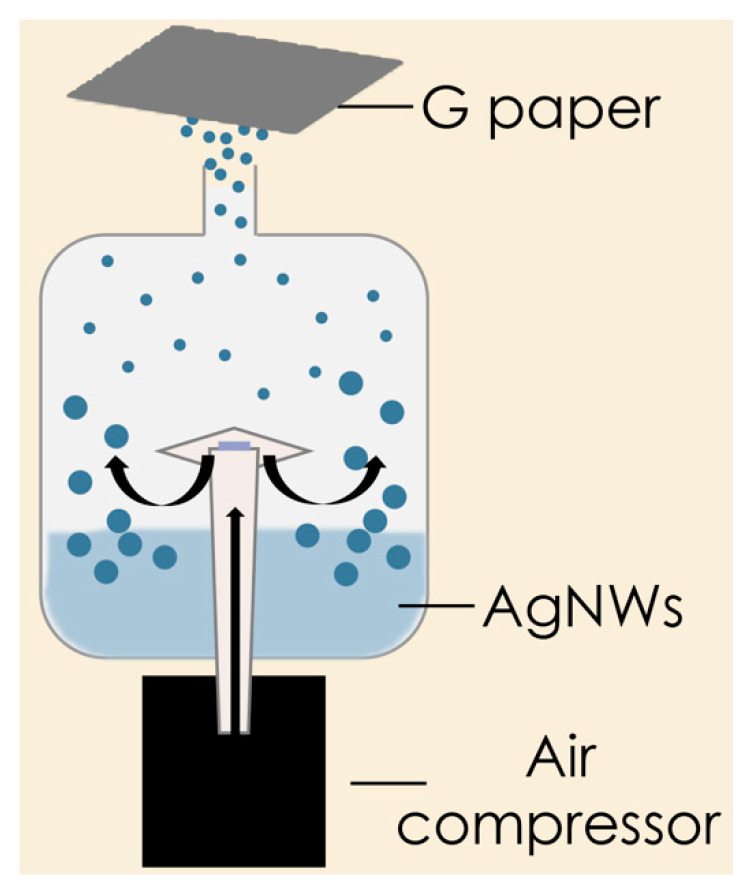
Scheme of aerosol deposition of AgNWs on G-paper.

Underlying our work was our established effort to create a substrate specifically designed to increase the local molecular density at plasmonic hotspots produced from a AgNWs network to maximize SERS signals from molecules typically showing a low Raman cross-section, as in References [12,14,15]. Briefly, we first adopted a standard wet chemistry procedure based on the polyol process to produce 5 ± 1 µm in length, 48 ± 10 nm in diameter AgNWs (Figure S1). These nanoparticles were receiving increasing consideration by the scientific community because of their large surface area and possibility to easily arrange them in bi- or tri-dimensional arrays [35]. Two-dimensional plasmonic substrates were then rapidly obtained by a nebulizing jet of a proper amount of AgNWs colloidal solution toward a 2 × 2 cm^2^ piece of thin graphene paper (G-paper) (Figure 1). The system included the possibility to impart a custom spacing of the graphene target to tune the covered airbrushed area, which was optimally set at 1 cm.

**Figure 2:**
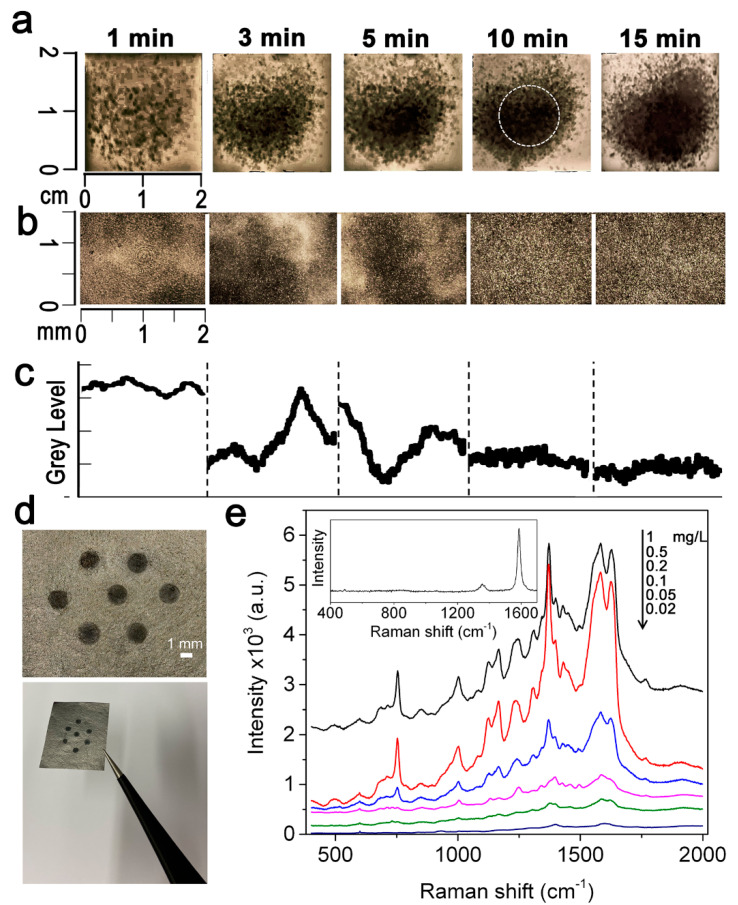
Fabrication of AgNWs@G-paper substrates. (**a**) Aerosol deposition of a 1 mg/L AgNW solution at different deposition times (varied from 1 min to 15 min) on a 2 × 2 cm^2^ piece of G-paper. The central 1 × 1 cm^2^ area of a AgNWs deposition showing a homogeneous density of deposited AgNWs (as highlighted by a dashed circle superposed on the sample at 10 min deposition) was considered for the following fabrication of patterned substrates (as in (**d**)); (**b**) Optical micrographs showing a 2 × 2 mm^2^ magnification of the central part of the substrates prepared in (**a**); (**c**) Grey level profiles showing the distribution of AgNWs within the areas considered in (**b**). A flat profile (as obtained after 10 min of AgNWs deposition) corresponds to a homogeneous AgNWs coating of G-paper; (**d**) Appearance of a AgNWs@G-paper substrate and a magnification of the AgNWs spot array as obtained by interposing a PET patterned mask between the nebulizing jet and the G-paper; (**e**) SERS spectra of Hb (1 × 10^−6^ M) on different AgNWs@G-paper substrates obtained by decreasing the density of nebulized AgNWs within the 1 ÷ 0.02 mg/L range once set the deposition time to 10 min (black, 1 mg/L; red, 0.5 mg/L; blue, 0.2 mg/L; purple, 0.1 mg/L; green, 0.05 mg/L; blue 0.02 mg/L). The 0.5 mg/L density provides the most intense Hb signals and was thus selected for further production of AgNWs@G-paper substrates. Spectra represent the average of over 20 acquisitions. Inset: Raman spectrum of G-paper.

In an attempt to create homogeneous AgNWs films, we varied the deposition time of the original AgNWs batch. A complete and homogeneous coating of the graphene support was obtained after 10 min of aerosol deposition (longer times did not further improve AgNWs coverage of the underlying graphene layer), while lower time values resulted in an uneven distribution (Figure 2a–c). Once we fixed the deposition time to 10 min, we varied the density of deposited AgNWs by sequential dilutions of the particle dispersion. In this case, a 10 mm-wide array of 1.5 mm in size silver spots was obtained by introducing a patterned mask consisting of a thin PET layer placed on the top of the G-paper once wetted with ethanol before nebulization, which ensured a temporary and a tight adhesion at the graphene/PET interface, in turn, avoiding possible edge-leakages of the nanoparticle solution. The mask was exactly centered with the graphene support (see the circled area in Figure 2a) to produce array spots at a comparable surface density of AgNWs. After deposition and mask removal, a pattern of regular and homogeneous AgNWs spots appeared well imprinted on the air-dried graphene (Figure 2d). Array spots obtained by 1:2 dilution of the AgNWs batch corresponding to a 0.5 mg/L density resulted to greatly enhance the Raman signal of 1 × 10^−6^ M hemoglobin (Hb) once excited at 532 nm (Figure 2e). We were able to detect the characteristic signals of Hb mainly ascribed to the heme group [36,37] (Table S1) down to 0.2-mg/L AgNWs density. After further dilution of deposited AgNWs, the protein signals progressively lost intensity, becoming undetectable at 0.02 mg/L where the spectrum corresponded to that of the underlying graphene (inset of Figure 2e). The above-optimized fabrication parameters were thus selected to optimally produce spotted AgNWs@G-paper substrates.

**Figure 3:**
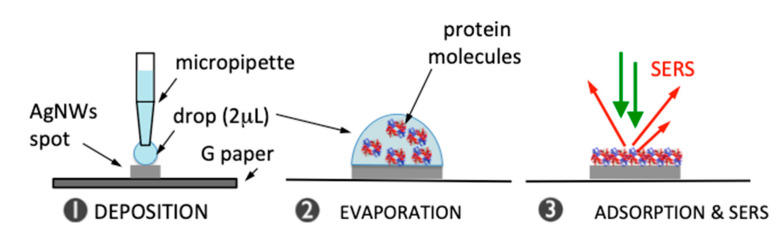
Working scheme of AgNWs@G-paper substrates: a small (2 µL) drop of protein solution is deposited on a AgNWs spot and confined due to the surrounding graphene hydrophobic barrier. After drop drying (~15 min at room temperature), the drop content is physically adsorbed on the surface of AgNWs and exposed to an effective E-field generating intense SERS signals.

We point out that the as-fabricated substrates allow to support the analysis of minimal quantities (2 µL) of biomolecule solution, which were initially dropped onto an array spot. They were then confined by a high contact angle formed with the surrounding hydrophobic graphene barrier (Figure S2) and finally effectively inspected under the Raman microscope once dried (Figure 3).

Our initial aim in choosing graphene as a low-cost support for nanoparticles was dictated by three main considerations: (1) superior hydrophobicity behavior with respect to other popular supports for disposable substrates as cellulose paper, thus enabling analyte confinement and concentration enrichment, as discussed above; (2) low Raman signals in the fingerprint region as compared to other candidate hydrophobic substrates as polytetrafluoroethylene (PTFE) and polydimethylsiloxane (PDMS) and limited to D (1354 cm^−1^) and G (1584 cm^−1^) band signals (Figure 2e inset); (3) additional features as quenching of autofluorescence signals frequently encountered in biomolecules [38] and better integration with biological entities, such as cells [39], for advanced biological analyses. With particular reference to the G-paper, further advantages were represented by easy-handling and easy-resizing, low-cost (0.2 €/cm^2^), and flexibility (with potential in the analysis of unflatten surfaces), which makes it even more attractive. 

**Figure 4:**
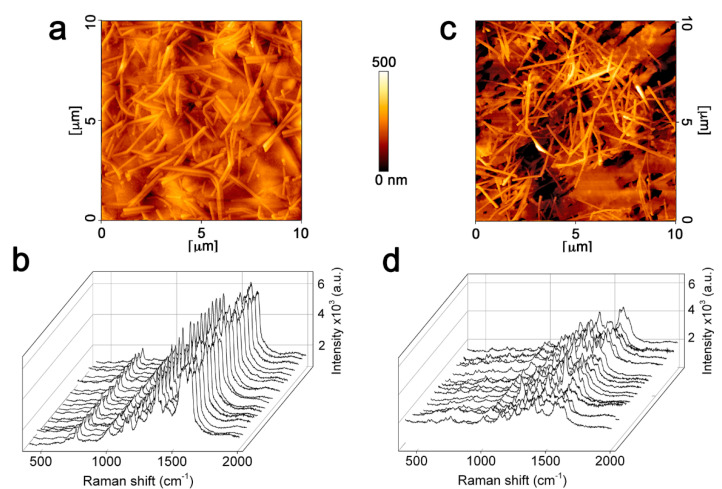
AFM topography of sprayed AgNWs on G-paper (**a**) and on a PTFE support (**c**). Random SERS spectra of Hb (1 × 10^−6^ M) as obtained by point-to-point mapping over 12 mm^2^ areas with a step size of 100 μm from sprayed AgNWs on G-paper (**b**) and on a PTFE support (**d**).

In the following, we showed that the choice of a graphene paper proved also advantageous in improving signal stability as well as in providing an ultra-flat background in comparison with other popular disposable supports for simple and rapid SERS analyses. The AFM investigation of AgNWs@G-paper revealed a homogeneous distribution of AgNWs on the micron scale (Figure 4a). The latter hypothesized a regular distribution of SERS hotspots, which was further demonstrated by inspecting the point-to-point signal of Hb over large areas (Figure 4b). A maximum relative standard deviation (RSD) <10% for the main Raman peaks of Hb was observed in this case. Conversely, when replacing G-paper with PTFE, AFM highlighted the presence of clustered wires unevenly covering the support surface (Figure 4c) and causing a larger point-to-point SERS signal variability (Figure 4d), which could be explained by a heterogeneous amplification of protein molecules [40]. G-paper played the role of catching interface [41] against sprayed wires, immobilizing them into a uniform surface distribution, which was not the case with plastic supports as PTFE. Furthermore, the high thermal and electrical conductivity of graphene [42] could contribute to buffering the laser radiation impact, generating well-resolved SERS spectra.

The amplification provided by the as-fabricated AgNWs@G-paper was evaluated by calculating the SERS enhancement factor (EF). EF is defined as the ratio between *I_Raman_* and *I_SERS_* normalized to the average number of molecules dispersed in solution *N_Raman_*, for the Raman measurement, and adsorbed onto the AgNWs hotspots *N_SERS_*, for the SERS measurement, respectively, which were present in the scattering volume (see SI for calculation of *N_SERS_* and *N_Raman_*):

$$EF=\frac{I_{SERS}/N_{SERS}}{I_{Raman}/N_{Raman}}$$

**Figure 5:**
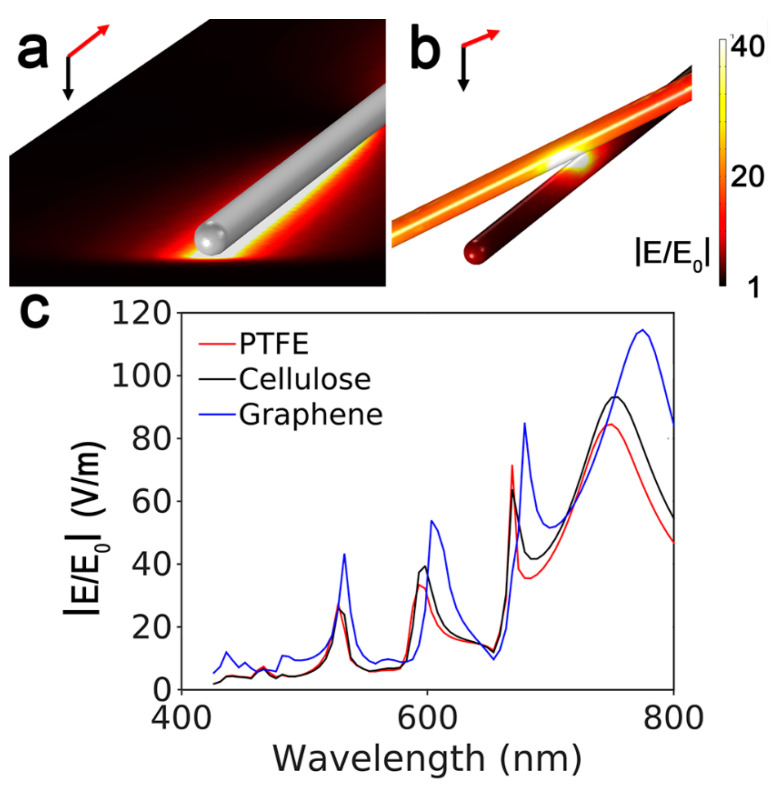
(**a**) FEM simulations of the E-field intensity in the proximity of a AgNW laying on a graphene surface. The E-field intensity is visualized for the plane corresponding to the G-paper surface while the wire is pictured as uniform grey color for better clarity. (**b**) The E-field intensity is visualized for two crossed AgNWs in the air. In this case, the E-field intensity on the wire surface is visualized. (**c**) Maximum E-field variation within the visible spectral range as simulated at the interfaces between a AgNW and graphene (blue), PTFE (red) and cellulose (black).

An *EF* value of 4 × 10^6^ was calculated, proving an enhancement ability of the same order of magnitude or higher than that from recently proposed low-cost disposable SERS substrates including those based on AgNWs assemblies [3,10,43,44,45]. We tried to gain further insights into the high-quality SERS profiles of AgNWs@G-paper by theoretical simulation of the electromagnetic field distribution. We may further note by AFM and optical analysis (Figure 4 and Figure S3) that the main part of AgNWs laid almost planar and in contact with graphene or forming single or few junctions with other AgNWs, suggesting the latter as the most representative configurations of the SERS enhancing capacity of AgNWs@G-paper substrates (Figure 5a,b and Figure S4). Nonetheless, the highest E-field values were found at the interfaces between AgNWs and graphene (Figure 5a). This is mostly not the case with planar SERS systems previously considered composed of AgNWs assemblies due to a higher wire density used, producing a large number of effective hotspots at crossed junctions [12,46,47]. The presence of graphene was instead supposed to favor AgNW/graphene with respect to AgNW/AgNW interactions, as discussed above (Figure 4a,b), leading to elongated (Figure 5a) hotspots providing a large space available for analyte accommodation and its detection. The higher average E-field value estimated at the graphene/AgNW interface hotspots, as compared to those formed at AgNWs interstitials ((|E|/|E_0_|)_graphene/AgNW_/(|E|/|E_0_|)_AgNW/AgNW_ = 1.2), could be explained by taking into account the higher refractive index of graphene in comparison to other popular supports, which boosts the near field within the gap between metal and graphene, as shown in Figure 5c. The near field value calculated at a graphene/AgNW interface under λ_Ex_ = 532 nm appears ∼2-fold larger (|E|/|E_0_| = 44) than that at a PTFE/AgNW (|E|/|E_0_| = 24) or cellulose/AgNWs (|E|/|E_0_| = 19) interfaces. As SERS enhancement is proportional to the fourth power of the E-field, an average *EF* = 3.7 × 10^6^ is obtained in the former case. A large matching between simulated and calculated *EF* let us hypothesize that the electromagnetic mechanism (EM) largely prevailed in AgNWs@G-paper substrates over a chemical mechanism (CM), as previously reported, as a possible additional effect in the SERS response of graphene-based substrates [48].

**Figure 6:**
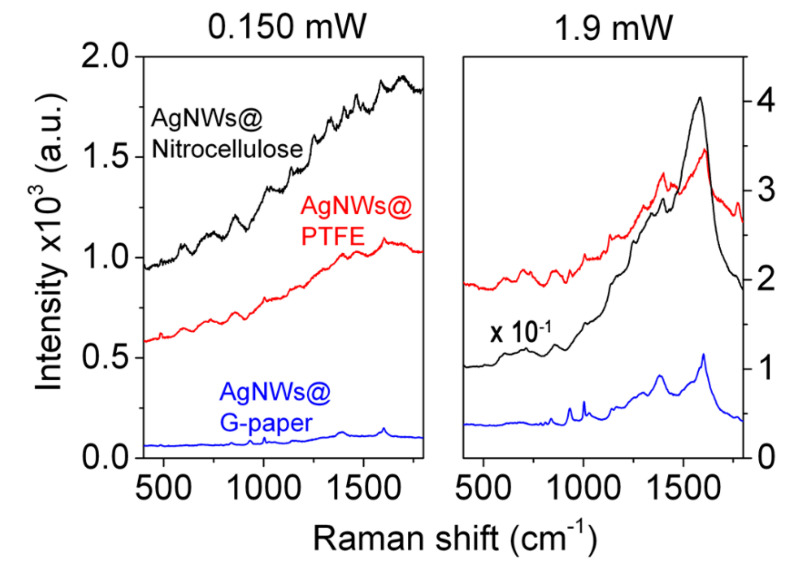
Background SERS profiles of AgNWs sprayed on nitrocellulose, PTFE and G-paper at (**left**) 0.150 mW and (**right**) 1.9 mW laser power at 532 nm. Spectra represent the average of over 20 acquisitions.

Another significant aspect in the choice of a SERS substrate resides in its background contribution to the overall SERS signal and generated at the interface between plasmonic layer and underlying support, which becomes critical especially in the detection of species with reduced SERS response, such as biomolecules. In Figure 6, a comparison between the background SERS signals of AgNWs on G-paper and that on popular substrates for disposable SERS substrates as nitrocellulose and PTFE is displayed. The background signal generated in the presence of G-paper remains the lowest regardless of the laser power employed and limited to the superposition between Raman modes of graphene and intrinsic signals pertaining to the AgNWs. On the opposite side, a fluorescence background governs the nitrocellulose and PTFE profiles (Figure 6 (left)), accompanied by the remarkable appearance of intense and broad amorphous carbon signals at 1350/1580 cm^−1^ [49] by the nitrocellulose-based substrate at high irradiation values (Figure 6 (right)), which affects partially or does not affect the signals of PTFE and G-paper, respectively (the latter linearly scaling with power). These results (1) depict graphene as a null fluorescence emitter due to its zero optical bandgap [50], generating an ultra-flat SERS background and (2) confirm the stability of graphene under more extreme power conditions and ascribed to its high thermal conductivity as above pointed out.

**Figure 7:**
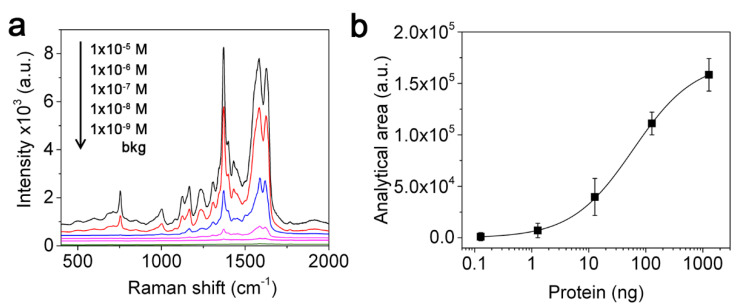
Detection sensitivity of the AgNWs@G-paper substrate. (**a**) SERS spectra of Hb ranging from 1 × 10^−5^ M to 1 × 10^−9^ M (black, 1 × 10^−5^ M; red, 1 × 10^−6^ M; blue, 1 × 10^−7^ M; violet, 1 × 10^−8^ M; purple, 1 × 10^−9^ M; green, background AgNWs@G-paper signal) corresponding to 1.3 μg to 0.3 ng of protein contained within 2 μL of analyzed sample volume. The background signal produced by the naked substrate is also displayed. (**b**) Correlation between the 1378 cm^−1^ band area of Hb and protein amounts (error bars represent the SD). Spectra and data points represent the average from 20 acquisitions.

We finally evaluated the detection sensitivity of the AgNWs@G-paper substrate by decreasing Hb concentration from 1 × 10^−5^ M to 1 × 10^−9^ M (1.3 μg to 0.3 ng) (Figure 7a). A sigmoid correlation (r^2^ > 0.99) between the band area of the 1378 cm^−1^ mode and the amount of protein was observed (Figure 7b), which can be commented on the one hand as a tendency to reach saturation on the available space of the hotspots at high-end values. On the other hand, a detection limit of 1 × 10^−8^ M was found, which corresponded to about 1 ng of protein in the analyzed sample volume (2 μL), suggesting a high sensitivity of AgNWs@G-paper that overcomes previous detection systems based on unsupported colloidal particles for protein detection including hemoglobin [51,52].

## 4. Conclusions

SERS substrates proposed so far are often unsuitable for the practical detection of biomolecules due to the lack of simultaneous presence of preferred characteristics, such as low manufacturing costs, disposable characteristics, and simplicity for routine use in turn limiting their use at or near point-of-need settings. In this work, we introduced a highly responsive SERS substrate relying on a simple aerosol deposition of AgNWs on graphene paper. The substrate was specifically designed for biodetection of small sample drops at submicromolar concentration. The proposed fabrication procedure relied on low-cost and facile steps that overcame a number of weak points frequently encountered in substrate preparation and patterning. These included the use of contaminating stabilizers to impart sufficient viscosity to nanoparticle inks as usually required in screen or ink-jet printing, as well as the need for dedicated instrumentation for top-down nanoparticle fabrication. Additionally, the presence of graphene improved the SERS response, conferring superior signal stability, low SERS background, and photostability. Overall, the proposed substrate exhibited high SERS efficiency, reliability, and sensitivity, as well as easy handling and usage aspects, meeting many of the requirements for effective and successful SERS detection of biomolecules. Future experiments on AgNWs@G-paper substrates will be aimed at exploring the SERS sensing of larger biological entities as cells for rapid and tag-free screening of tumor diseases (e.g., malignant cells) or to monitor light treatments (e.g., laser-exposed cells) for therapeutic applications. 

## Data Availability

Data are contained within the article and supplementary material.

## Supplementary Materials

The following are available online at https://www.mdpi.com/article/10.3390/nano11061495/s1, Figure S1: (a) SEM image and (b) UV-vis spectrum of the AgNWs used to prepare the SERS substrates, Table S1: Assignment of main SERS signals of Hb, Figure S2: Contact angle measurement of water on AgNWs@graphene; Figure S3: Optical image of an AgNWs@Gpaper substrate; Figure S4: FEM simulation of a AgNW laying on graphene and surmounted by another crossed wire.

## References

[1] LeRu E.C., Etchegoin P.G. (2009). Principles of Surface-Enhanced Raman Spectroscopy: And Related Plasmonic Effects.

[2] Aroca R. (2006). Surface-Enhanced Vibrational Spectroscopy.

[3] Oliveira M.J., Quaresma P., De Almeida M.P., Araujo A., Pereira E., Fortunato E., Martins R., Franco R., Aguas H. (2017). Office paper decorated with silver nanostars—An alternative cost effective platform for trace analyte detection by SERS. Sci. Rep..

[4] Banchelli M., De Angelis M., D’Andrea C., Pini R., Matteini P. (2018). Triggering molecular assembly at the mesoscale for advanced Raman detection of proteins in liquid. Sci. Rep..

[5] Mu Y.Y., Zhang X.P. (2020). A paper-fiber-supported 3D SERS substrate. Plasmonics.

[6] Lee M., Oh K., Choi H.K., Lee S.G., Youn H.J., Lee H.L., Jeong D.H. (2018). Subnanomolar sensitivity of filter paper-based SERS sensor for pesticide detection by hydrophobicity change of paper surface. ACS Sens..

[7] Yu W.W., White I.M. (2010). Inkjet printed surface enhanced raman spectroscopy array on cellulose paper. Anal. Chem..

[8] Yang Q., Deng M.M., Li H.Z., Li M.Z., Zhang C., Shen W.Z., Li Y.N., Guo D., Song Y.L. (2015). Highly reproducible SERS arrays directly written by inkjet printing. Nanoscale.

[9] Qu L.L., Li D.W., Xue J.Q., Zhai W.L., Fossey J.S., Long Y.T. (2012). Batch fabrication of disposable screen printed SERS arrays. Lab A Chip.

[10] Wu W., Liu L., Dai Z.G., Liu J.H., Yang S.L., Zhou L., Xiao X.H., Jiang C.Z., Roy V.A.L. (2015). Low-cost, disposable, flexible and highly reproducible screen printed sers substrates for the detection of various chemicals. Sci. Rep..

[11] Park S.G., Mun C., Lee M., Jeon T.Y., Shim H.S., Lee Y.J., Kwon J.D., Kim C.S., Kim D.H. (2015). 3D Hybrid plasmonic nanomaterials for highly efficient optical absorbers and sensors. Adv. Mater..

[12] Banchelli M., Amicucci C., Ruggiero E., D’Andrea C., Cottat M., Ciofini D., Osticioli I., Ghini G., Siano S., Pini R. (2019). Spot-on SERS detection of biomolecules with laser-patterned dot arrays of assembled silver nanowires. ChemNanoMat.

[13] Bruzas I., Lum W., Gorunmez Z., Sagle L. (2018). Advances in surface-enhanced Raman spectroscopy (SERS) substrates for lipid and protein characterization: Sensing and beyond. Analyst.

[14] Barucci A., D’Andrea C., Farnesi E., Banchelli M., Amicucci C., De Angelis M., Hwang B., Matteini P. (2021). Label-free SERS detection of proteins based on machine learning classification of chemo-structural determinants. Analyst.

[15] Banchelli M., Cascella R., D’Andrea C., Cabaj L., Osticioli I., Ciofini D., Li M.S., Skupien K., De Angelis M., Siano S. (2020). Nanoscopic insights into the surface conformation of neurotoxic amyloid beta oligomers. RSC Adv..

[16] Gen M.S., Chan C.K. (2017). Electrospray surface-enhanced Raman spectroscopy (ES-SERS) for probing surface chemical compositions of atmospherically relevant particles. Atmos. Chem. Phys..

[17] Benedetti D.P., Zhang J., Tague T.J., Lombardi J.R., Leona M. (2014). In situ microanalysis of organic colorants by inkjet colloid deposition surface-enhanced Raman scattering. J. Raman Spectrosc..

[18] Fang H., Zhang X., Zhang S.J., Liu L., Zhao Y.M., Xu H.J. (2015). Ultrasensitive and quantitative detection of paraquat on fruits skins via surface-enhanced Raman spectroscopy. Sens. Actuators B—Chem..

[19] Han D.L., Li B.X., Chen Y., Wu T., Kou Y.C., Xue X.J., Chen L., Liu Y., Duan Q. (2019). Facile synthesis of Fe3O4@Au core-shell nanocomposite as a recyclable magnetic surface enhanced Raman scattering substrate for thiram detection. Nanotechnology.

[20] Brayner R., Iglesias R., Truong S., Beji Z., Felidj N., Fievet F., Aubard J. (2010). Surface-enhanced raman scattering on silver nanostructured films prepared by spray-deposition. Langmuir.

[21] Li B.W., Zhang W., Chen L.X., Lin B.C. (2013). A fast and low-cost spray method for prototyping and depositing surface-enhanced Raman scattering arrays on microfluidic paper based device. Electrophoresis.

[22] Demirta O., Doganay D., Ozturk I.M., Unalan H.E., Bek A. (2020). Facile preparation of nanoparticle based SERS substrates for trace molecule detection. Phys. Chem. Chem. Phys..

[23] Yang G.H., Fang X.J., Jia Q., Gu H.X., Li Y.P., Han C.Q., Qu L.L. (2020). Fabrication of paper-based SERS substrates by spraying silver and gold nanoparticles for SERS determination of malachite green, methylene blue, and crystal violet in fish. Microchim. Acta.

[24] Jang W., Byun H., Kim J.H. (2020). Rapid preparation of paper-based plasmonic platforms for SERS applications. Mater. Chem. Phys..

[25] Zhou Y.Z., Cheng X.N., Du D., Yang J., Zhao N., Ma S.B., Zhong T., Lin Y.H. (2014). Graphene-silver nanohybrids for ultrasensitive surface enhanced Raman spectroscopy: Size dependence of silver nanoparticles. J. Mater. Chem. C.

[26] Zhou Y.Z., Cheng X.N., Yang J., Zhao N., Ma S.B., Li D., Zhong T. (2013). Fast and green synthesis of flexible free-standing silver nanoparticles-graphene substrates and their surface-enhanced Raman scattering activity. RSC Adv..

[27] Liu J., Liu L.B., Wu X.W., Zhang X.K., Li T.D. (2015). Environmentally friendly synthesis of graphene-silver composites with surface-enhanced Raman scattering and antibacterial activity via reduction with L-ascorbic acid/water vapor. N. J. Chem..

[28] Li Y.T., Qu L.L., Li D.W., Song Q.X., Fathi F., Long Y.T. (2013). Rapid and sensitive in-situ detection of polar antibiotics in water using a disposable Ag-graphene sensor based on electrophoretic preconcentration and surface-enhanced Raman spectroscopy. Biosens. Bioelectron..

[29] Liu M.M., Chen W. (2013). Graphene nanosheets-supported Ag nanoparticles for ultrasensitive detection of TNT by surface-enhanced Raman spectroscopy. Biosens. Bioelectron..

[30] Waxenegger J., Trugler A., Hohenester U. (2015). Plasmonics simulations with the MNPBEM toolbox: Consideration of substrates and layer structures. Comput. Phys. Commun..

[31] Rakic A.D., Djurisic A.B., Elazar J.M., Majewski M.L. (1998). Optical properties of metallic films for vertical-cavity optoelectronic devices. Appl. Opt..

[32] Zhu Y.W., Murali S., Cai W.W., Li X.S., Suk J.W., Potts J.R., Ruoff R.S. (2010). Graphene and graphene oxide: Synthesis, properties, and applications. Adv. Mater..

[33] Novotny L. (2007). Effective wavelength scaling for optical antennas. Phys. Rev. Lett..

[34] Rossouw D., Botton G.A. (2013). Plasmonic response of bent silver nanowires for nanophotonic subwavelength waveguiding. Phys. Rev. Lett..

[35] Kwon J., Suh Y.D., Lee J., Lee P., Han S., Hong S., Yeo J., Lee H., Ko S.H. (2018). Recent progress in silver nanowire based flexible/wearable optoelectronics. J. Mater. Chem. C.

[36] Rusciano G., De Luca A.C., Pesce G., Sasso A. (2008). Raman tweezers as a diagnostic tool of hemoglobin-related blood disorders. Sensors.

[37] Wood B.R., Tait B., McNaughton D. (2001). Micro-Raman characterisation of the R to T state transition of haemoglobin within a single living erythrocyte. Biochim. Biophys. Acta Mol. Cell Res..

[38] Xie L.M., Ling X., Fang Y., Zhang J., Liu Z.F. (2009). Graphene as a substrate to suppress fluorescence in resonance raman spectroscopy. J. Am. Chem. Soc..

[39] Jasim D.A., Lozano N., Bussy C., Barbolina I., Rodrigues A.F., Novoselov K.S., Kostarelos K. (2018). Graphene-based papers as substrates for cell growth: Characterisation and impact on mammalian cells. Flatchem.

[40] Matteini P., Cottat M., Tavanti F., Panfilova E., Scuderi M., Nicotra G., Menziani M.C., Khlebtsov N., De Angelis M., Pini R. (2017). Site-selective surface-enhanced raman detection of proteins. ACS Nano.

[41] Xu W.G., Mao N.N., Zhang J. (2013). Graphene: A platform for surface-enhanced raman spectroscopy. Small.

[42] Sang M., Shin J., Kim K., Yu K.J. (2019). Electronic and thermal properties of graphene and recent advances in graphene based electronics applications. Nanomaterials.

[43] Yu W.W., White I.M. (2012). A simple filter-based approach to surface enhanced Raman spectroscopy for trace chemical detection. Analyst.

[44] Shi Y.E., Li L.M., Yang M., Jiang X.H., Zhao Q.Q., Zhan J.H. (2014). A disordered silver nanowires membrane for extraction and surface-enhanced Raman spectroscopy detection. Analyst.

[45] Wang Q., Zhao X.C., Yu Z.N., Tan R.Q., Lan J. (2015). Large scale preparation of surface enhanced Raman spectroscopy substrates based on silver nanowires for trace chemical detection. Anal. Methods.

[46] Tao A.R., Yang P.D. (2005). Polarized surface-enhanced Raman spectroscopy on coupled metallic nanowires. J. Phys. Chem. B.

[47] Jang S., Lee J., Nam S., Ko H., Chang S.T. (2017). Large-area, highly sensitive SERS substrates with silver nanowire thin films coated by microliter-scale solution process. Nanoscale Res. Lett..

[48] Lai H.S., Xu F.G., Zhang Y., Wang L. (2018). Recent progress on graphene-based substrates for surface-enhanced Raman scattering applications. J. Mater. Chem. B.

[49] Heck C., Kanehira Y., Kneipp J., Bald I. (2019). Amorphous carbon generation as a photocatalytic reaction on DNA-assembled gold and silver nanostructures. Molecules.

[50] Zheng P., Wu N.Q. (2017). Fluorescence and sensing applications of graphene oxide and graphene quantum dots: A review. Chem. -Asian J..

[51] Xu L.J., Zong C., Zheng X.S., Hu P., Feng J.M., Ren B. (2014). Label-free detection of native proteins by surface-enhanced Raman spectroscopy using iodide-modified nanoparticles. Anal. Chem..

[52] Kahraman M., Balz B.N., Wachsmann-Hogiu S. (2013). Hydrophobicity-driven self-assembly of protein and silver nanoparticles for protein detection using surface-enhanced Raman scattering. Analyst.

