# Supplementary material for: Cost Effective Silver Nanowire-Decorated Graphene Paper for Drop-On SERS Biodetection
Source: Nanomaterials (Basel). 2021 Jun 4;11(6):1495. doi: 10.3390/nano11061495 (PMC8229787; doi:10.3390/nano11061495)
Supplement: Supplementary file 1 [file nanomaterials-11-01495-s001.zip › nanomaterials-1227958-supplementary.pdf]

nanomaterials

Supplementary Material

Cost Effective Silver Nanowire-Decorated Graphene Paper for Drop-on SERS Biodetection

Chiara Amicucci [1,2], Cristiano D'Andrea [1], Marella de Angelis [1], Martina Banchelli [1], Roberto Pini and Paolo Matteini [1,*]

[1] "Nello Carrara" Institute of Applied Physics (IFAC) of the Italian National Research Council (CNR), Via Madonna del Piano 10, 50019 Sesto Fiorentino, Italy; chiara.amicucci@unifi.it (C.A.); c.dandrea@ifac.cnr.it (C.D.); m.deangelis@ifac.cnr.it (M.d.A.); m.banchelli@ifac.cnr.it (M.B.); r.pini@ifac.cnr.it (R.P.)
[2] Department of Industrial Engineering, University of Florence, Via Santa Marta 3, 50134 Florence, Italy
* Correspondence: p.matteini@ifac.cnr.it

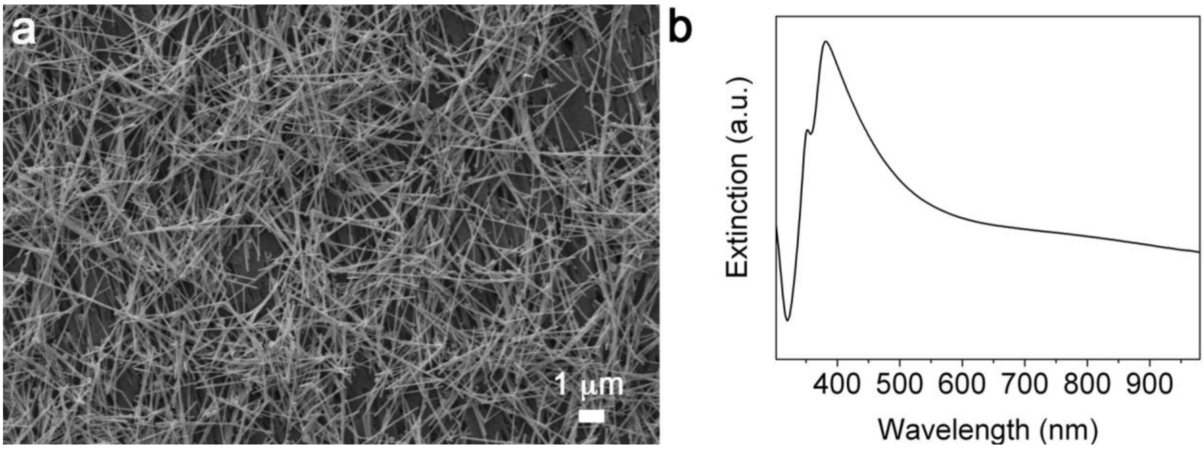

Figure S1. (**a**) SEM image and (**b**) UV-vis spectrum of the AgNWs used to prepare the SERS substrates.

Table S1. Assignment of main SERS signals of Hb.

**Assignment**<br>**λ_{Ex} = 532 nm**	
680	ν_{7}
750	ν_{15}
1123	ν_{22}
1161	ν_{30}
1230	ν_{13}
1309	ν_{21}
1378	ν_{4}
1393	ν_{20}
1430	ν_{28}
1555	ν_{11}
1582	ν_{37}
1610	ν_{19}
1627	ν_{10}

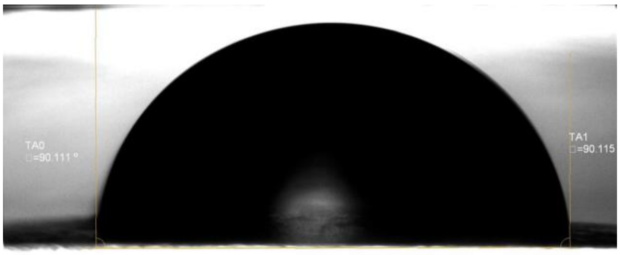

Figure S2. Contact angle of a 2 µL water drop on a AgNWs@graphene spot.

EF calculation

EF was calculated for the AgNWs@G-paper substrate at the optimal 0.1 µg/mm^2 AgNW density. A 2-µl drop of Hb (1×10^{-6} M) was dried on a single spot of a laser-patterned AgNWs@PTFE substrate and immediately analysed. SERS maps at λ_{Ex} = 532 nm were collected from 3 replica substrates and averaged. Raman measurements on a 1×10^{-3} M Hb solution in water in quartz cuvette (1 cm) were also performed. The EF was calculated by comparing the 750 cm^{-1} band intensity of Hb in the SERS spectrum (I_{SERS}) with that of the Raman measurement (I_{Raman}) according to the equation:

$$EF = \frac{I_{SERS}/N_{SERS}}{I_{Raman}/N_{Raman}} \quad \text{(S.1)}$$

where

$$N_{Raman} = C_{molecule} \times V_f \times N_A = C_{molecule} \times A_{waist} \times H_{depth} \times N_A \quad \text{(S.2)}$$

being $C_{molecule}$ the concentration of Hb solution used for Raman measurements, V_f the focal volume, A_{waist} the laser spot area in the focal plane, H_{depth} the depth-of-field of the objective (10x) and N_A the Avogadro number,

$$N_{SERS} = D_{AgNWs} \times A_{waist} \times {}^{A_{eff}}\!/_{f_p} \quad \text{(S.3)}$$

being D_{AgNWs} the AgNW density on G-paper, A_{eff} the effective area of the hotspot accounting for 98% of the signal, and f_p the footprint area of Hb molecule.

Therefore

$$\frac{N_{Raman}}{N_{SERS}} = \frac{C_{molecule}\times A_{waist}\times H_{depth}\times N_A}{D_{AgNWs}\times A_{waist}\times {}^{A_{eff}}\!/_{f_p}} = \frac{C_{molecule}\times H_{depth}\times N_A\times f_p}{D_{AgNWs}\times A_{eff}} \quad \text{(S.4)}$$

with H_{depth} = 216 µm at 532 nm, f_p = 20 nm^2/molecule for Hb, D_{AgNWs} = 1.6 AgNWs/µm^2 and A_{eff} = 5×10^4 nm^2. I_{SERS}/I_{Raman} is 113 and thus the EF value is 4×10^6.

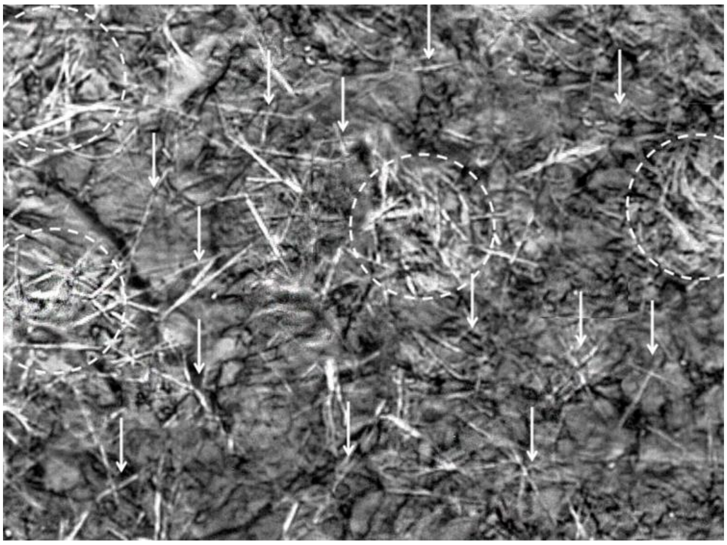

Figure S3. Optical image (90µm × 65µm) of an AgNWs@G-paper substrate. Arrows: isolated AgNWs or forming single junctions with other AgNWs; circles: AgNWs forming more than a single junction with other AgNWs.

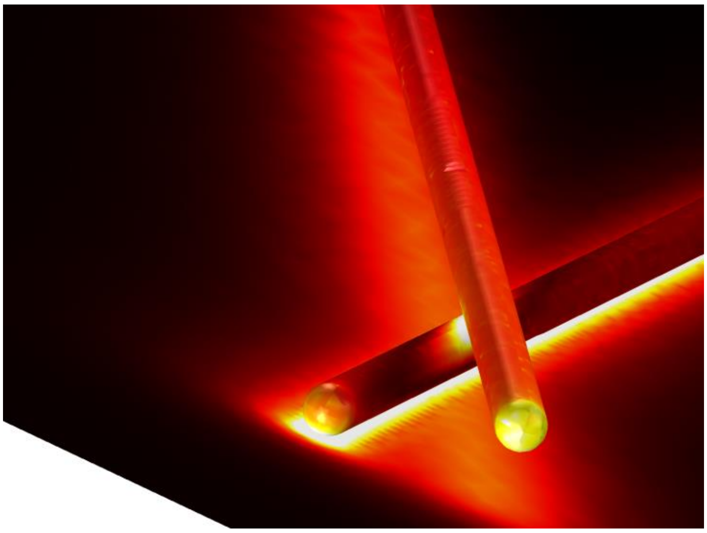

Figure S4. FEM simulation of a AgNW laying on graphene and surmounted by another crossed wire. SERS hotspots at the graphene/AgNW interface and at the AgNW/AgNW interstitial appear spatially confined. The ratio between the average E-fields values calculated within these hotspots approaches that found by comparing the values of the same hotspots in separate configurations as displayed in Figures. 5a,b.
